# Pathological response following neoadjuvant immune checkpoint inhibitors in patients with hepatocellular carcinoma: a cross-trial, patient-level analysis

**DOI:** 10.1016/S1470-2045(24)00457-1

**Published:** 2024-10-19

**Authors:** Antonio D’Alessio, Bernardo Stefanini, Julia Blanter, Benjamin Adegbite, Fionnuala Crowley, Vincent Yip, Sarah Slater, Claudia Angela Maria Fulgenzi, Ciro Celsa, Giulia Francesca Manfredi, Madhava Pai, Robert D Goldin, Stephen C Ward, Maria Isabel Fiel, Daniel H Shu, Yung-Yeh Su, Alessio Cortellini, Marina Baretti, Robert Anders, Mark Yarchoan, Chiun Hsu, Thomas U Marron, David J Pinato

**Affiliations:** Division of Cancer, Department of Surgery and Cancer, Imperial College London, London, UK; Division of Cancer, Department of Surgery and Cancer, Imperial College London, London, UK; Department of Medical and Surgical Sciences, University of Bologna, Bologna, Italy; Department of Medicine, Division of Hematology–Oncology, Tisch Cancer Institute, Mount Sinai Hospital, New York, NY, USA; Department of Medicine, Division of Hematology–Oncology, Tisch Cancer Institute, Mount Sinai Hospital, New York, NY, USA; Department of Medicine, Division of Hematology–Oncology, Tisch Cancer Institute, Mount Sinai Hospital, New York, NY, USA; Barts and The London HPB Centre, Barts Health NHS Trust, London, UK; Department of Medical Oncology, Barts Health NHS Trust, London, UK; Division of Cancer, Department of Surgery and Cancer, Imperial College London, London, UK; Division of Cancer, Department of Surgery and Cancer, Imperial College London, London, UK; Gastroenterology and Hepatology Unit, Department of Health Promotion, Mother & Child Care, Internal Medicine & Medical Specialties, University of Palermo, Palermo, Italy; Division of Cancer, Department of Surgery and Cancer, Imperial College London, London, UK; Department of Translational Medicine, Università del Piemonte Orientale “A. Avogadro”, Novara, Italy; Division of Surgery, Department of Surgery and Cancer, Imperial College London, London, UK; Department of Digestive Diseases, Imperial College London, St Mary’s Hospital, London, UK; Department of Pathology, Molecular and Cell-Based Medicine, Icahn School of Medicine at Mount Sinai, New York, NY, USA; Department of Pathology, Molecular and Cell-Based Medicine, Icahn School of Medicine at Mount Sinai, New York, NY, USA; Sidney Kimmel Comprehensive Cancer Center, Johns Hopkins University School of Medicine, Baltimore, MD, USA; National Institute of Cancer Research, National Health Research Institutes, Tainan, Taiwan; Department of Oncology, National Cheng Kung University Hospital, College of Medicine, National Cheng Kung University, Tainan, Taiwan; Department of Internal Medicine, Kaohsiung Medical University Hospital and Center for Cancer Research, Kaohsiung Medical University, Kaohsiung, Taiwan; Division of Cancer, Department of Surgery and Cancer, Imperial College London, London, UK; Operative Research Unit of Medical Oncology, Fondazione Policlinico Universitario Campus Bio-Medico, and Department of Medicine and Surgery, Universitá Campus Bio-Medico di Roma, Rome, Italy; Sidney Kimmel Comprehensive Cancer Center, Johns Hopkins University School of Medicine, Baltimore, MD, USA; Sidney Kimmel Comprehensive Cancer Center, Johns Hopkins University School of Medicine, Baltimore, MD, USA; Sidney Kimmel Comprehensive Cancer Center, Johns Hopkins University School of Medicine, Baltimore, MD, USA; Department of Medical Oncology, National Taiwan University Cancer Center, Taipei, Taiwan; Department of Oncology, National Taiwan University Hospital, Taipei, Taiwan; Department of Medicine, Division of Hematology–Oncology, Tisch Cancer Institute, Mount Sinai Hospital, New York, NY, USA; Division of Cancer, Department of Surgery and Cancer, Imperial College London, London, UK; Department of Translational Medicine, Università del Piemonte Orientale “A. Avogadro”, Novara, Italy

## Abstract

**Background:**

Neoadjuvant use of immune checkpoint inhibitors (ICIs) before liver resection results in pathological tumour regression in patients with hepatocellular carcinoma. We aimed to describe the characteristics of pathological responses after preoperative ICI therapy for hepatocellular carcinoma and to evaluate the association between the depth of tumour regression and relapse-free survival.

**Methods:**

In this cross-trial, patient-level analysis, we performed a pooled analysis of data from patients with hepatocellular carcinoma receiving ICI therapy before liver resection as part of a global collaborative consortium (NeoHCC) of five phase 1 and 2 clinical trials and standardised observational protocols conducted in 12 tertiary referral centres across the USA, UK, and Taiwan. Eligible patients were adults (aged ≥18 years) diagnosed with hepatocellular carcinoma by tissue core biopsy before treatment initiation, a Liver Imaging Reporting and Data System score of 5 on imaging, or both, with an Eastern Cooperative Oncology Group performance status score of 0–1, and no extrahepatic spread or previous ICI treatment. Pathological response was measured as the percentage of non-viable tumour in the resected surgical specimen, with major pathological response corresponding to at least 70% tumour regression and pathological complete response corresponding to 100% tumour regression. We correlated pathological response with radiological overall response using RECIST criteria (version 1.1) and relapse-free survival, and evaluated the threshold of tumour regression that could be optimally associated with relapse-free survival.

**Findings:**

At data cutoff on Jan 31, 2024, 111 patients were included in the study, of whom data on pathological response were available for 104 (94%) patients. Patients received treatment from Oct 5, 2017, to Nov 15, 2023, mostly ICI combinations (76 [69%]), for a median of 1·4 months (IQR 0·7–2·9). 87 (78%) patients were men and 24 (22%) were women. Most patients had underlying viral chronic liver disease (73 [66%]) and Barcelona Clinic Liver Cancer stage A hepatocellular carcinoma (61 [55%]), without portal vein thrombosis (87 [78%]). We observed major pathological response in 33 (32%) patients and pathological complete response in 19 (18%) patients. Radiological overall response was associated with major pathological response, with 23 (74%) of 31 patients with radiological response showing major pathological response compared with ten (14%) of 73 patients without radiological response (p<0·0001). However, ten (30%) of 33 major pathological responses were not predicted by radiological response. After a median follow-up of 27·2 months (95% CI 22·3–32·1), median relapse-free survival for the whole cohort was 43·6 months (95% CI 28·3–not evaluable). Relapse-free survival was significantly longer in patients with major pathological response than in those who did not have a major pathological response (not reached [95% CI not evaluable–not evaluable] *vs* 28·3 months [12·8–43·8]; hazard ratio 0·26 [0·10–0·66]; p=0·0024) and in patients with pathological complete response than in those who did not have a pathological complete response (NR [95% CI not evaluable–not evaluable] *vs* 32·8 months [15·0–50·5]; 0·19 [0·05–0·78]; p=0·010). Unbiased recursive partitioning of the cohort for the risk of relapse, death, or both identified a threshold of 90% as the optimal cutoff of pathological tumour regression to predict improved relapse-free survival.

**Interpretation:**

The extent of tumour regression following neoadjuvant ICI therapy could identify patients with improved relapse-free survival following liver resection. The threshold of at least 90% tumour regression should be validated for its surrogate role for relapse-free survival in phase 3 randomised controlled trials.

**Funding:**

None.

## Introduction

Hepatocellular carcinoma is a global health challenge, with more than 830 000 deaths per year and a predicted increase in incidence and mortality of over 50% in the next two decades.^1^ Immune checkpoint inhibitors (ICIs) have become the standard of care for patients with unresectable or metastatic hepatocellular carcinoma, with atezolizumab–bevacizumab and durvalumab–tremelimumab approved for routine first-line clinical use.^2,3^ For patients with early-stage hepatocellular carcinoma, liver resection might lead to cure; however, relapse rates can be as high as 70% in the first 5 years following resection.^4^ There is an acute need to establish optimal use of ICIs alongside curative therapies, such as resection or ablation. Clinical data have shown that, compared with surveillance, inhibitors of the PD-1 pathway with or without bevacizumab can lead to an improvement in relapse-free survival after liver resection.^5,6^

Preoperative use of ICIs in patients with hepatocellular carcinoma has been explored in several small, early-phase studies, in which heterogeneous therapeutic combinations were tested, mainly with safety and feasibility being the primary endpoints assessed.^7–12^ Clinical evidence of resectable stage IIIB to IVC malignant melanoma suggests that neoadjuvant immunotherapy is superior to adjuvant treatment alone in improving relapse-free survival following resection.^13^ Accumulating translational evidence supports the view that neoadjuvant ICI therapy might lead to increased immune cell activation mediated by the presence of the primary tumour in situ, including expansion of the T-cell clone pool and the immune reconstitution of exhausted tumour-specific lymphocytes.^14^

Evolving experience from the use of systemic anticancer therapy in solid tumours suggests that a pathologically demonstrated response in resected specimens predicts improved chances of complete remission in patients with various oncological diagnoses, including breast cancer, non-small-cell lung cancer, and bladder cancer.^15–17^ However, due to the small sample size of the individual clinical trials conducted in the neoadjuvant setting of hepatocellular carcinoma to date, it is not clear whether the observation of pathologically demonstrable tumour regression translates into long-term survival benefit.

This study is the first to assess a global cohort of patient-level data from patients with hepatocellular carcinoma receiving neoadjuvant ICI therapy. We aimed to describe the characteristics of pathological responses after preoperative ICI therapy in patients with hepatocellular carcinoma and to evaluate the association between the depth of tumour regression and relapse-free survival.

## Methods

### Study design and participants

NeoHCC is a global collaborative consortium collecting patient-level data from patients with hepatocellular carcinoma receiving neoadjuvant ICI therapy before liver resection. This consortium is part of five prospective phase 1 and 2 clinical trials and standardised observational protocols being conducted in 12 tertiary referral centres across the USA, UK, and Taiwan. Protocols of the individual studies, including schedules of events, types of agents used, and the participant inclusion and exclusion criteria adopted for each trial, have been described previously.^7,9,11,12,18^

All patients were adults (aged ≥18 years) diagnosed with hepatocellular carcinoma by tissue core biopsy before treatment initiation or a Liver Imaging Reporting and Data System (LI-RADS) score of 5 on imaging, or both, as per the criteria of the American Association for the Study of Liver Diseases.^19^ All patients clustered within Child–Pugh class A liver function criteria, with an Eastern Cooperative Oncology Group performance status score of 0–1. Eligibility criteria included the presence of active measurable disease as per Response Evaluation Criteria in Solid Tumours (RECIST) criteria (version 1.1)^20^ at study baseline. Before neoadjuvant treatment, tumours were considered to be upfront resectable or potentially resectable after successful downstaging. Patients with extrahepatic disease, contraindications to ICI, or a history of solid organ transplantation were excluded.

The study was conducted according to the ethics guidelines in the Declaration of Helsinki. Ethical approval to conduct this study was granted following review of each individual study protocol by the individual ethics committee of each participating site. Written, informed patient consent was obtained for participation in each individual prospective study. Collection and analysis of fully anonymised clinical data were conducted as part of a research protocol approved by Imperial College Tissue Bank (reference number R15058).

### Procedures

Anonymised data on the patients’ demographics were collected from electronic medical records and their clinical status was updated following the publication of primary trial results at every participating site. Data on ethnicity were not available for all individual studies and therefore were not presented. All patients received treatment regimens containing ICI therapy before undergoing liver resection.^7,9,11,12,18^ ICIs administered in the studies included monoclonal antibodies targeting PD-1 (nivolumab and cemiplimab), PD-L1 (atezolizumab), and CTLA-4 (ipilimumab). Postoperative adjuvant ICI monotherapy was administered in one of the studies for a planned duration of 6 months.^7^ Each clinical trial mandated the assessment of a pathological response as part of their primary^7^ or secondary efficacy endpoints,^9,11^ and this assessment was prospectively collected and reported as part of the exploratory aims for all cohorts.^12,18^ Pathological response was measured as the percentage of tumour bed occupied by non-viable tumour on the surgical resection specimen after the completion of the neoadjuvant ICI course, following international reporting guidance (appendix pp 4, 18).^21^

### Statistical analysis

We investigated the association between the depth of pathological response and both relapse-free survival and time to relapse using Cox regression models. The proportional hazards assumption was verified with scaled Schoenfeld residuals. Relapse-free survival was defined as the time from the date of the first dose of neoadjuvant ICI to the date of radiological evidence of tumour relapse, death, or last follow-up assessment, whichever occurred first. Time to relapse was defined as the time from the date of the first dose of neoadjuvant ICI to the date of radiological evidence of tumour relapse or last follow-up assessment, whichever occurred first.

We described the distribution of pathological response by subdividing the cohort into numerically homogeneous quartiles on the basis of the grade of pathological response. We considered major pathological response as the presence of at least 70% of a non-viable tumour, as per previous publications,^7,8^ and pathological complete response as the complete absence of residual viable tumour. The 70% cutoff was extrapolated from previous studies on pathological responses to transarterial chemoembolisation,^22^ but has not been validated in multiple independent cohorts. We explored the role of a different cutoff for response using unbiased recursive partitioning to predict the risk of relapse, death, or both, and the receiver operating characteristic (ROC) curve for relapse-free survival over 24 months. We chose 24 months on the basis of historical cohorts, given that it captured more than 50% of relapses in patients who had undergone liver resection.^4^ We used the non-parametric method for the ROC curve analysis because it does not assume any specific distribution of the test results and is robust across different types of data distributions.^23^

Radiological response was assessed as per RECIST criteria on CT or MRI 3–6 weeks after the start of ICI therapy, according to individual protocols. Modified RECIST criteria were used to assess response in a subset of patients.^24^ Radiological overall response rate was considered to be the sum of the rates of complete response and partial response, whereas disease control rate included the rates of complete response, partial response, and stable disease. Overall survival was defined as the time from the date of the first dose of the treatment to the date of death or last follow-up, whichever occurred first.

Baseline characteristics were summarised as categorical or continuous variables, as appropriate, and reported with descriptive statistics. We investigated the association between the occurrence of a major pathological response and baseline characteristics, including the treatment regimen received. Fisher’s exact test or Pearson’s χ^2^ test were used to compare nominal variables, with Fisher’s exact test preferred for 2 × 2 contingency tables with small sample sizes (less than five expected counts) and Pearson’s χ^2^ test used for other cases. A Student’s *t* test was used to compare the parametric distribution of continuous variables across categories, whereas the Mann–Whitney test was used for non-parametric distribution. We used linear regression to evaluate the association between two continuous variables (*R*^2^ coefficient). Overall survival, time to relapse, and relapse-free survival were estimated with the Kaplan–Meier method, including survival rates at 1 and 2 years, and compared with the log-rank test, whereas median follow-up was calculated with the reverse Kaplan–Meier method. We also included a survival analysis restricted only to patients not receiving adjuvant therapy to exclude the potential confounding role of post-operative ICI therapy. We further tested the independent prognostic value of different variables in association with relapse-free survival by multivariable analysis using Cox regression models with the log likelihood ratio test. We avoided the risk of collinearity in the multivariable model by excluding overlapping variables (eg, portal vein tumoral thrombosis and Barcelona Clinic Liver Cancer [BCLC] stage). Collinearity was tested with the variance inflation factor, calculated with the *olsrr* R package.

Acknowledging the potential cross-trial variability in assessing tumour regression, a centre-specific conditional interpretation with use of frailty models was applied to correct the 95% CIs from the Cox regressions. For the same reason, a cluster correction per different centre was applied to the multivariable model. ROC curves were used to assess the prognostic ability of pathological response in predicting patients’ relapse-free survival at the 24-month endpoint. We used the *pROC* R package to estimate the sensitivity and specificity of different response thresholds, and we used the Youden’s index (J) to calculate the optimal major pathological response value that maximised the true-positive rate and minimised the false-positive rate (J=specificity + sensitivity − 1).^23^ The Youden’s index was weighted for the occurrence of major pathological response in the cohort and for the relative cost of false negatives and false positives. We performed an unbiased recursive partitioning for the risk of relapse or death as validation of the optimal cutoff for response. Additionally, we built a further conditional inference tree adjusted per treating centre and treatment modality. All p values were two-sided, and confidence intervals were set at the 95% level, with significance predefined to be at less than 0·05. All statistical analyses were performed with IBM SPSS Statistics (version 28.0), R (version 4.3.1) or Rstudio (2023.09.1), and GraphPad Prism (version 10.0).

### Role of the funding source

There was no funding source for this study.

## Results

**Figure 1:**
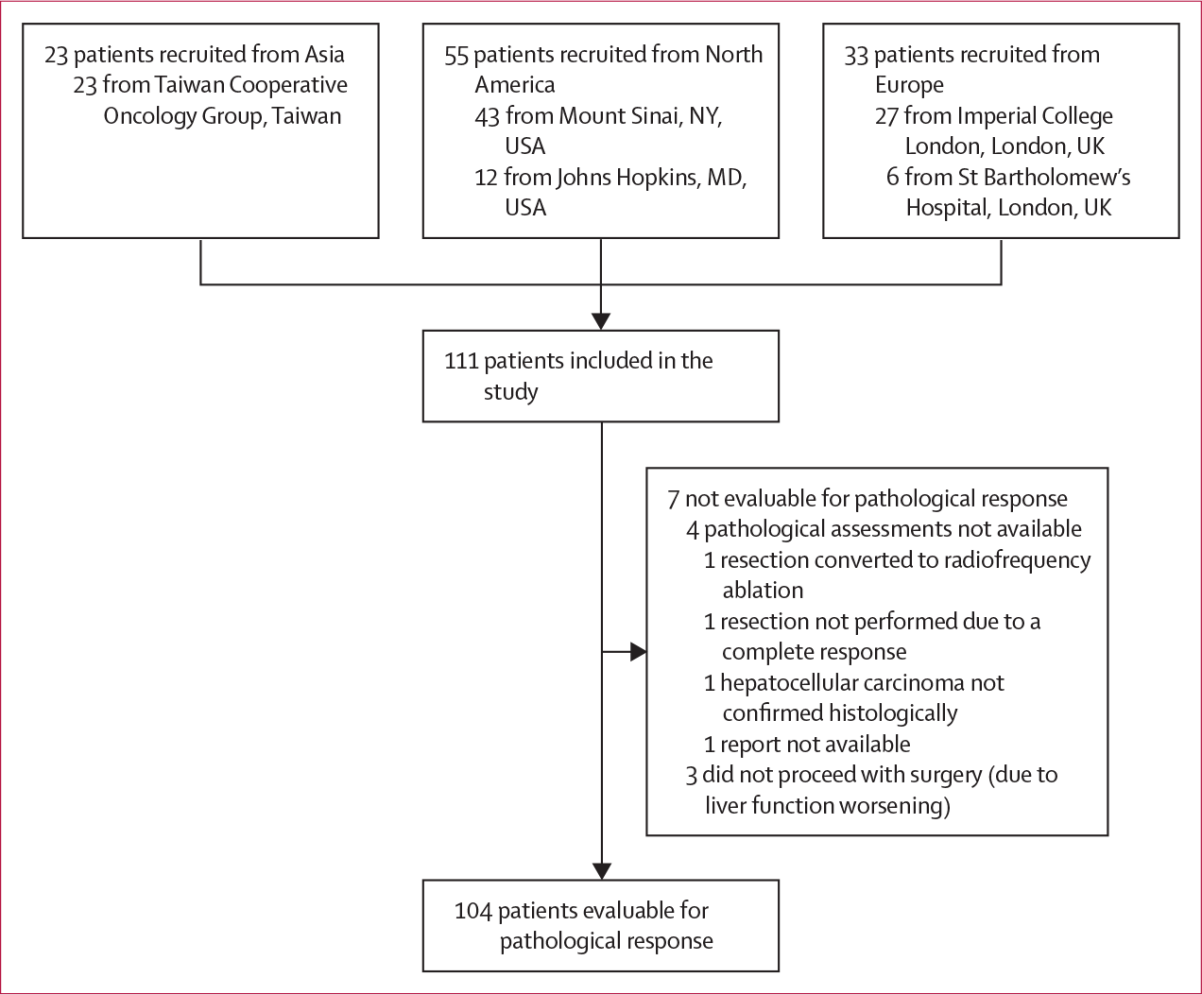
Study profile

At data cutoff on Jan 31, 2024, 111 patients were included in the study. Patients were treated from Oct 5, 2017, to Nov 15, 2023, at Mount Sinai Hospital, New York, NY, USA (43 patients [39%]); Imperial College London, London, UK (27 [24%]); Taiwan Cooperative Oncology Group, Taiwan (23 [21%]); Johns Hopkins University, Baltimore, MD, USA (12 [11%]), and St Bartholomew’s Hospital, London, UK (six [5%]; figure 1; appendix p 13). 87 (78%) patients were men and 24 (22%) were women, and most had an Eastern Cooperative Oncology Group performance status score of 0 (94 [85%]), and viral-related hepatocellular carcinoma (73 [66%]), of whom 40 (36%) patients had hepatitis B virus, 31 (28%) had hepatitis C virus, and two (2%) had coinfection with hepatitis B and C virus (table 1). 51 (46%) patients had a histological diagnosis of liver cirrhosis and 83 (75%) patients had an available fibroscan assessment before surgery, with 15 (18%) individuals with stage F0, nine (11%) with F1, nine (11%) with F2, six (7%) with F3, and 44 (53%) with F4. All patients had preserved liver function and were Child–Pugh class A. Diagnosis of hepatocellular carcinoma was histologically confirmed with a tissue core biopsy before treatment initiation in 94 (85%) patients, with the remaining 17 (15%) patients diagnosed with radiological LI-RADS criteria, which was confirmed on the surgical specimen after completion of ICI therapy. At baseline, most tumours were staged as BCLC stage A (61 [55%]) or B (26 [23%]), with presence of portal vein tumoral thrombosis in 24 (22%) patients and no extrahepatic spread in any patient. For patients with portal vein tumoral thrombosis, Vp assessment was available in 13 (51%) patients, with six (46%) individuals classified as Vp1, three (23%) as Vp2, two (15%) as Vp3, and two (15%) as Vp4. 21 (19%) patients had received at least one previous local therapy, most of whom had received one therapy (14 patients [13%]), including transarterial chemoembolisation (ten [9%]), surgery (seven [6%]), radiofrequency ablation (five [5%]), radioembolisation (three [3%]), and stereotactic body radiotherapy (one [1%]).

Anti-PD-1 monotherapy was administered in 35 (32%) patients, whereas the remaining 76 (68%) patients were treated with combination therapy, including double checkpoint inhibition with anti-PD-1–anti-CTLA-4 (57 [51%]), tyrosine kinase inhibitor–anti-PD-1 (12 [11%]), and anti-VEGF–anti-PD-L1 (seven [6%]). In 17 (15%) patients, an adjuvant course of anti-PD-1 monotherapy was administered, as per trial protocol,^7^ for a median of 5·5 months (IQR 3·7–6·0).

Median duration of neoadjuvant treatment was 1·4 months (IQR 0·7–2·9), and median time between the commencement of neoadjuvant therapy and liver resection was 2·3 months (1·4–3·3). All patients were assessed for radiological response after the completion of neoadjuvant treatment. 31 (28%) patients had a radiological overall response, including four (4%) patients with a complete response and 27 (24%) patients with a partial response. Overall disease control was observed in 101 (91%) patients and ten (9%) patients had primary progression. Radiological response was assessed with modified RECIST criteria in 81 (71%) patients, of whom a radiological overall response was reported in 26 (32%) patients, a complete response in five (6%) patients, and disease control in 77 (95%) patients (appendix p 14).

For the whole cohort, median follow-up was 27·2 months (95% CI 22·3–32·1). Relapse, death, or both occurred in 40 (36%) patients. Median relapse-free survival of the whole cohort was 43·6 months (95% CI 28·3–not evaluable [NE]; appendix p 5). The relapse-free survival rate was 82% (95% CI 75–90) at 1 year and 61% (52–73) at 2 years. With only 13 (12%) deaths recorded in the cohort, median overall survival was not mature at the time of the analysis and could not been estimated (appendix p 5). The overall survival rate was 97% at 1 year (95% CI 94–100) and 89% (83–96) at 2 years.

**Figure 2:**
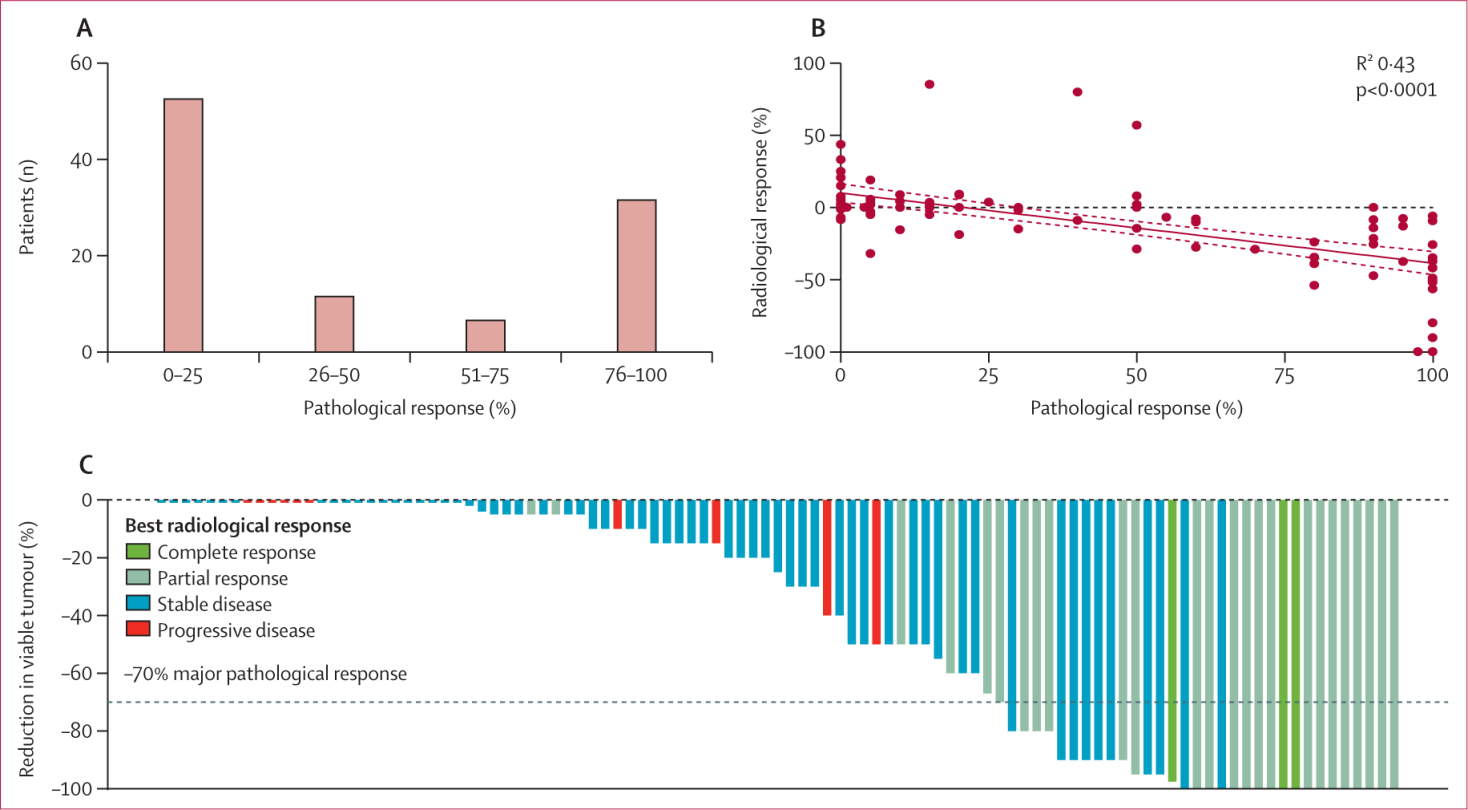
Pathological responses of surgical samples from patients after neoadjuvant immunotherapy and association with radiological responses (A) Pathological responses assessed in the surgical samples by percentage of tumour regression (n=104). (B) The correlation between change in tumour size based on radiological assessment (RECIST criteria [version 1.1]) and pathological response of each surgical specimen. (C) Waterfall plot for the depth of pathological response, with different bar colours according to radiological response as per RECIST criteria (version 1.1); the dashed line represents major pathological response (70% tumour regression). RECIST=Response Evaluation Criteria in Solid Tumours.

The individual percentage of pathological responses was available for 104 (94%) of 111 patients. We observed a major pathological response in 33 (32%) pathologically evaluable patients and a pathological complete response in 19 (18%). The distribution of individual grades of pathological responses was bimodal, with 85 (82%) of the 104 samples clustering either in the group showing little to no response (53 [51%]) or in the group with more than 75% of response (32 [31%]). 19 (18%) samples had a response between 25% and 75%, with 12 (12%) clustering within the 26–50% group and seven (7%) within the 51–75% group (figure 2A). The individual percentages of pathological response and radiological response seemed to be significantly correlated (*R*^2^ 0·43; p<0·0001; figure 2B). However, when categorising pathological response according to the occurrence of a major pathological response (figure 2C), we noticed a partial association with radiological response. Although patients with either a partial response or complete response as best radiological response were five times more likely to have a major pathological response than were those with no radiological response (23 [74%] of 31 *vs* ten [14%] of 73; p<0·0001), ten (30%) of 33 patients had a major pathological response in the absence of a radiological response. However, with the use of modified RECIST criteria, the discrepancy between major pathological response and radiological overall response rate decreased to 17%: of 23 patients with a major pathological response with an available modified RECIST assessment, four (17%) did not show a radiological response, whereas only five (10%) of 51 patients without a major pathological response showed a radiological response per modified RECIST criteria.

The occurrence of a major pathological response was not associated with any relevant baseline clinical characteristics, except for the presence of cirrhosis, with 20 (61%) of 33 patients with a major pathological response having cirrhosis compared with 26 (37%) of 71 patients who did not have a major pathological response (p=0·022; table 2). Rates of major pathological response did not differ between pathologically evaluable patients receiving combination therapy and those receiving ICI monotherapy (26 [38%] of 69 *vs* seven [20%] of 35; p=0·067). We assessed differences in major pathological response rates between patients receiving double ICI combination therapy and those receiving ICI plus an antiangiogenic agent, and did not find a significant association (18 [36%] of 50 *vs* eight [42%] of 19; p=0·78; appendix p 6).

**Figure 3:**
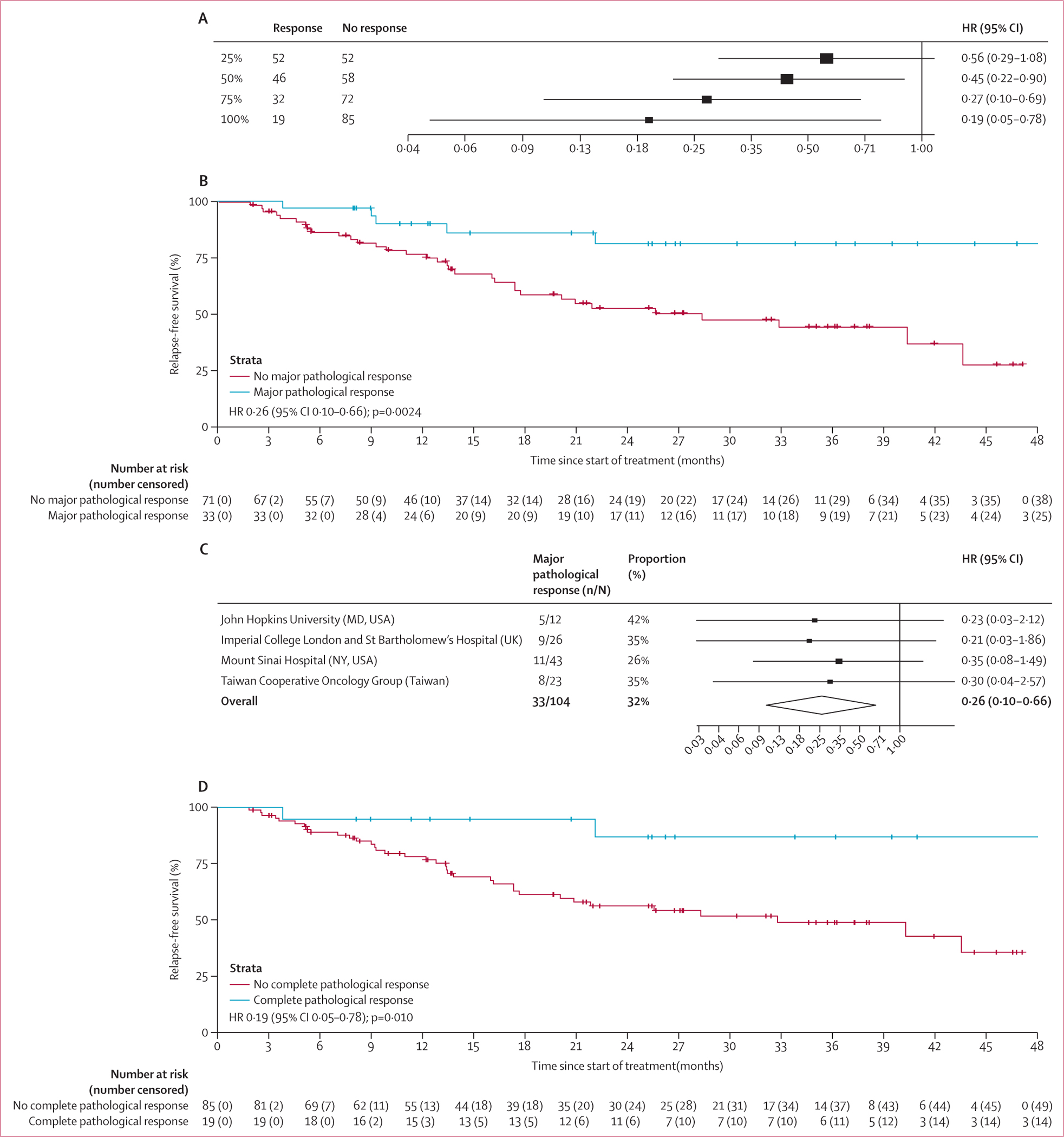
Association between major pathological response and pathological complete response and relapse-free survival (A) Forest plot representing the HRs for relapse-free survival associated with different thresholds of pathological responses in the whole cohort (n=104). (B) Kaplan–Meier curves for relapse-free survival according to the occurrence of major pathological response; vertical dashed denote censored patients (C). Forest plot showing the association between the occurrence of major pathological response and improvement in relapse-free survival within each cohort of the NeoHCC study consortium; the size of the shapes (squares and diamond) denote effects size. Patients with available pathological assessment data were included (n=104). Imperial College London and St Bartholomew’s Hospital were part of the same clinical trial (NCT03682276).^12^ Patients treated at Mount Sinai Hospital within the clinical trial^8^ or the observational clinical study^20^ were centrally assessed for pathological response and data were pooled together. (D) Kaplan–Meier curves for relapse-free survival according to the occurrence of pathological complete response; vertical dashed denote censored patients. HR=hazard ratio.

When considering pathological response as a continuous variable, we observed a significant association between the depth of response and improved relapse-free survival (hazard ratio [HR] 0·990 [95% CI 0·981–0·998; p=0·018). We proceeded to explore the relationship between relapse-free survival and different thresholds of pathological response and found that the HR decreased progressively with the increase in the cutoff for response (figure 3A).

To describe the characteristics of pathological regression observed in resected samples, we divided the percentages of tumour regression into the following quartiles: 25 (24%) patients with 0% (quartile 1), 27 (26%) patients with 1–25% (quartile 2), 24 (23%) patients with 26–90% (quartile 3), and 28 (27%) patients with 91–100% (quartile 4). We observed an increase in relapse-free survival rates over 24 months with increasing quartiles (quartile 1: 60%; quartile 2: 64%; quartile 3: 65%; and quartile 4: 82%), supporting the association between the degree of tumour response and improvement in relapse-free survival.

We then focused on major pathological response, defined as the presence of at least 70% of tumour regression identified in the surgical specimen. 33 patients had a major pathological response and 71 did not. Patients with a major pathological response had significantly improved median relapse-free survival compared with those who did not have a major pathological response (not reached [95% CI NE–NE] *vs* 28·3 months [12·8–43·8]; HR 0·26 [95% CI 0·10–0·66]; p=0·0024; figure 3B). We addressed the potentially confounding role of adjuvant treatment by performing a further survival analysis only in patients who had not received adjuvant ICI (94 [85%] patients), and found that major pathological response was significantly associated with relapse-free survival (HR 0·23 [0·09–0·60]; p=0·0011; appendix p 7). The association between major pathological response and relapse-free survival was observed within all cohorts participating in the study, despite the 95% CIs crossing 1 in the individual cohorts as a likely consequence of the small sample size of the single studies (figure 3C), and it was also investigated according to the presence of portal vein tumoural thrombosis, BCLC stage, and treatment modality (appendix p 8). When applying a cluster correction to account for the effect of different ICI regimens and potentially different pathological assessments within each centre, major pathological response remained independently associated with relapse-free survival, even after adjusting for baseline clinical characteristics (appendix p 15). The proportional hazards assumption was verified for each variable and for the model as a whole (appendix p 9). In particular, the presence of cirrhosis, which was found to be associated with major pathological response rates, was not shown to be an independent predictor of relapse-free survival, alongside other baseline factors including sex, cause, performance status, BCLC stage, fibrosis, portal vein thrombosis, tumour diameter, and treatment modality, with the only exception being previous local therapy (appendix pp 10, 16). The HR and the 95% CIs of the association between major pathological response and relapse-free survival did not change after applying a frailty model for each individual centre (HR 0·26 [95% CI 0·10–0·66]; p=0·0024). The occurrence of a major pathological response was significantly associated with a reduced risk of relapse at 24 months, with five (15%) of 33 patients with a major pathological response having relapsed or died at 2 years compared with 28 (39%) of 71 patients who did not have a major pathological response (p=0·014; appendix p 17). The only baseline factor associated with an increased risk of relapse at 24 months was the receipt of previous locoregional treatment, whereas no association was found with other baseline characteristics, including sex, performance status, BCLC stage, cause, portal vein thrombosis, cirrhosis, and grade of fibrosis (appendix p 17).

Aiming to identify the optimal threshold of pathological response as a predictor of relapse-free survival, we performed an unbiased recursive partitioning of the whole cohort for the risk of relapse, death, or both. We identified that the ideal cutoff for pathological response was 90%, which remained unaltered after adjusting for individual centre and treatment modality (monotherapy or combination ICI therapy; appendix p 11). We applied Youden’s index on the ROC curve for the relapse-free survival at 24 months. We estimated that the optimal response threshold to predict for relapse, death, or both at 2 years was 92·5%, with a sensitivity of 93·8% and a specificity of 29·0%. The threshold of 70% used to define major pathological response was found to have a sensitivity of 84·4% and a specificity of 39·1%. Median relapse-free survival was significantly improved in patients reporting a pathological complete response, with an HR of 0·19 (95% CI 0·05–0·78) favouring pathological complete response (median relapse-free survival not reached [95% CI NE–NE]) over no pathological complete response (32·8 months [15·0–50·5]; p=0·010; figure 3D).

Relapse occurred in 36 (32%) patients. For the whole cohort, median time to relapse was estimated to be 43·6 months (95% CI 32·8–NE). Patients with a major pathological response reached a significantly improved median time to relapse compared with those who did not have a major pathological response (not reached [95% CI NE–NE] *vs* 32·8 months [16·9–48·7]; HR 0·23 [0·08–0·65; p=0·0024). Time to relapse was significantly associated with the percentage of pathological regression (HR 0·989 [0·980–0·998]; p=0·016). Ten (14%) patients who did not have a major pathological response and three (9%) patients with a major pathological response died. Death was not attributed to toxicity related to ICI therapy in any patient, and occurred at a median of 17·2 months (IQR 12·3–23·8) after the end of systemic therapy. Death was associated with disease progression in nine (69%) patients and with septic shock in one patient, cardiac myocardial infarction in one patient, worsening of liver function in one patient, and sudden death at home from an unknown cause in one patient. Although the data on median overall survival were not mature at the time of data cutoff, an initial evaluation of the association between major pathological response or pathological complete response is shown in the appendix (p 12).

## Discussion

The use of ICIs as a neoadjuvant treatment before liver resection for patients with hepatocellular carcinoma has been explored in several single-centre phase 1 and 2 clinical trials. Collectively, these pioneering studies have shown the safety and feasibility of a perioperative ICI treatment approach.^7–12,25^ However, none of these studies were individually powered to robustly investigate predictive factors of efficacy from neoadjuvant ICI therapy, particularly with regards to long-term survival outcomes, including relapse-free survival. This study from the NeoHCC consortium was designed to overcome this issue by pooling prospectively collected patient-level data from phase 1 and 2 clinical trials run in a consortium of tertiary referral centres.^7,9,11,12^ To our knowledge, NeoHCC is the first study to show a statistically significant and clinically meaningful relationship between the percentage of non-viable tumour after ICI therapy and a proportional improvement in relapse-free survival following liver resection among patients with hepatocellular carcinoma.

In this study, the magnitude of improvement in relapse-free survival increased from a HR of 0·56 in patients with at least 25% tumour regression to a HR of 0·19 in those with a pathological complete response. 23 (82%) of the 28 patients with near-total tumour regression (91–100%) were alive and free from relapse at 24 months, compared with 15 (60%) of 25 patients without evidence of pathological response. Although this study did not include a prospectively enrolled control group, it should be emphasised that patients treated with liver surgery alone in historical cohorts showed a risk of recurrence exceeding 50% at the 24-month timepoint,^4^ underscoring the contribution of neoadjuvant ICI in reducing relapse rates.

Interestingly, we observed that the depth of pathological response followed a bimodal distribution, with 85 (82%) of 104 samples showing either a response close to pathological complete response (>75%) or little to no response (<25%), leaving only 19 (18%) samples with intermediate degrees of response (26–75%). These findings are in line with what has been previously shown in patients with non-small-cell lung cancer, whereby a population of patients with early-stage disease undergoing neoadjuvant chemotherapy combined with ICI showed a similar polarisation of pathological responses.^16^ Molecular classifications have identified classes of hepatocellular carcinoma associated with ICI responsiveness (inflamed class) and ICI resistance (excluded class).^26^ Future studies should focus on the association between the molecular features of the tumour microenvironment and the depth of pathological response.

Success from neoadjuvant ICI therapy is strongly reliant on the extent of T-cell-mediated immune rejection, resulting in tumour necrosis. A key purpose of this NeoHCC study was to understand whether the extent of necrosis—ie, a surrogate of treatment-induced restoration of effector immune-cell function within the tumour microenvironment—might be associated with a postoperative risk of recurrence and death. To achieve this aim, we stratified patients based on those who showed at least 70% tumour regression on the surgical specimen after ICI therapy, defined as a major pathological response.

Patients with a major pathological response showed a significantly longer relapse-free survival than did patients who did not have a major pathological response, independently of baseline characteristics or type of treatment received. Considering that most cases of hepatocellular carcinoma arise following a history of chronic liver impairment,^3^ we recognised that relapse-free survival might be potentially affected by deaths caused by hepatic decompensation.^27^ To specifically identify the relationship between pathological response and the risk of disease relapse, we conducted an exploratory analysis of time to relapse, which was also found to be significantly associated with tumour regression.

Alongside pathological response, the clinical trials included in this study consortium also measured the efficacy of neoadjuvant ICI in terms of radiological response. Interestingly, we observed only a partial association between radiological response and pathological response. Although patients with a radiological overall response had a significantly increased chance of having a major pathological response, we observed that the presence of a major pathological response was not predicted by a radiological response in almost a third of patients, thus suggesting that the use of radiological endpoints might underestimate the actual benefit from ICI therapy. These findings mirror the evidence from a pooled analysis of neoadjuvant trials in patients with other oncological indications, in which up to 27% of patients without an radiological overall response were found to have a major pathological response on surgical specimens.^28^ The reason for this discrepancy might lie in the mechanism of action of ICIs, which induce unique patterns of response mediated by T cells within the tumour microenvironment without necessarily affecting the tumour size. In the specific context of hepatocellular carcinoma response, the integration of modified RECIST criteria in radiological assessments might be helpful in better capturing tumour necrosis induced by ICI therapy in future trial designs.

One of the key questions is whether major pathological response can be adopted as a valid surrogate for improvement in relapse-free survival. This study provides the first cross-trial investigation aimed at establishing the optimal threshold of pathological response for predicting relapse-free survival. Clinical studies have been inconsistent with the choice of a cutoff of major pathological response, with some studies adopting 70%^7,8,25^ and other studies adopting 90%,^9,10^ and the 70% threshold has not received multicentre validation. In other cancer types, the endpoint to define response to neoadjuvant therapy has been pathological complete response (100%)^13,15^ or near-pathological complete response (>90%).^29^ We performed an unbiased recursive partitioning for relapse-free survival and found that the optimal cutoff of pathological response to predict the risk of relapse, death, or both in our cohort was 90%, further validating this cutoff with Youden’s index. In view of the likely upcoming era of phase 3 randomised trials for neoadjuvant ICI therapy in patients with hepatocellular carcinoma, it will be of the utmost importance to reach a shared, global consensus on the threshold of response as an endpoint to assess the efficacy of ICIs in this setting.

Although our findings indicate enhanced relapse-free survival among patients with a major pathological response, those without a pathological response showed a significantly increased risk of relapse. More than two thirds of patients achieved less than 70% tumour regression, including 25 (24%) patients showing no regression at all. Aside from its important prognostic role, major pathological response might potentially refine patient selection for treatment following surgery. Based on our results and the relatively small benefit of relapse-free survival conferred by adjuvant therapy,^6^ adjuvant ICI treatment might be preferentially reserved to patients without sufficient tumour regression during neoadjuvant therapy, sparing patients with a complete response from unnecessary and potentially toxic treatment.

We acknowledge several limitations in our study. This analysis included patients with heterogeneous tumour presentations, ranging from resectable BCLC stage A tumours to those with upfront unresectable tumours. The variety of ICI-based treatments and their durations prompted us to substantiate the independent association between pathological response and relapse-free survival within each separate cohort. However, we cannot exclude the possibility that specific regimens might have differential effects on the occurrence of a major pathological response or relapse-free survival benefit. Additionally, the different timing of response assessment across study protocols might have affected the association between radiological overall response and major pathological response. Despite including the largest cohort of patients treated with neoadjuvant ICI therapy for hepatocellular carcinoma to date, the sample size of this study consortium did not allow a definitive confirmation of surrogacy; therefore, this should be investigated in prospective, randomised, phase 3 trials that include a comparator group. Furthermore, the small number of patients precluded internal random cross-validation of our recursive partitioning models due to the inability to generate adequately sized training and validation cohorts. Another limitation lies in the absence of central histological review, despite the fact that the interobserver agreement has been shown to be consistently high among experienced pathologists in assessing pathological response in patients with other cancer types.^30^ Acknowledging the potential interobserver variability in the assessment of pathological response, we applied frailty models to the Cox regression analyses and included a cluster correction for each different centre in the multivariable model. Finally, although tumour immune infiltrate has been described in detail in the individual studies included in this analysis,^7,9,18,25^ the inherently clinical aim of this study prevented us from pooling together the translational data from the different cohorts to investigate cross-trial biomarkers of ICI therapy efficacy. This investigation will be the focus of future research by this consortium.

In conclusion, the NeoHCC study represents the first global benchmark for the characterisation of pathological response to neoadjuvant ICI therapy in patients with hepatocellular carcinoma. In the largest pooled prospective analysis to date, we were able to show at a patient level that pathological response to neoadjuvant ICI in hepatocellular carcinoma is a robust predictor of improved relapse-free survival, underscoring its potential as a clinical endpoint for future, randomised, phase 3 trials.

## Supplementary Material

Supplement Appendix

## Figures and Tables

**Table 1:** Baseline patient characteristics

	Patients (n=111)

Age, years	67 (38–81)
Sex	
Male	87 (78%)
Female	24 (22%)
Eastern Cooperative Oncology Group performance status score	
0	94 (85%)
1	17 (15%)
Barcelona Clinic Liver Cancer stage	
A	61 (55%)
B	26 (23%)
C	24 (22%)
Cause of disease	
HBV	40 (36%)
HCV	31 (28%)
HBV-HCV coinfection	2 (2%)
Alcohol	11 (10%)
Metabolic-associated fatty liver disease	6 (5%)
Cryptogenic	21 (19%)
Cirrhosis	
Present	51 (46%)
Absent	60 (54%)
Portal vein thrombosis	
Present	24 (22%)
Absent	87 (78%)
Previous local therapy	
No	90 (81%)
1	14 (13%)
2	5 (5%)
3	2 (2%)
Tumour diameter, cm	6·0 (3·9–9·9)
Number of nodules	1 (1–2)
Neoadjuvant regimen	
Anti-PD-1 monotherapy	35 (32%)
Anti-CTLA4-anti-PD-1	57 (51%)
Tyrosine kinase inhibitor-anti-PD-1	12 (11%)
Anti-VEGF-anti-PD-L1	7 (6%)
Adjuvant therapy	
Yes	17 (15%)
No	94 (85%)

Data are median (range) for age, median (IQR) for tumour diameter and number of nodules, and n (%) for all other variables. HBV=hepatitis B virus. HCV=hepatitis C virus.

**Table 2:** Baseline patient characteristics by occurrence of major pathological response

	Major pathological response (n=33)	No major pathological response (n=71)	p value

Age, years	64 (47–80)	67 (38–81)	0·45
Sex			
Male	27 (82%)	54 (76%)	0·51
Female	6 (18%)	17 (24%)	··
Eastern Cooperative Oncology Group performance status score			
0	27 (82%)	62 (87%)	0·55
1	6 (18%)	9 (13%)	··
Barcelona Clinic Liver Cancer stage			
A	13 (39%)	44 (62%)	0·10
B	10 (30%)	14 (20%)	··
C	10 (30%)	13 (18%)	··
Cause of disease			
Viral	25 (76%)	46 (65%)	0·26
Non-viral	8 (24%)	25 (35%)	··
Portal vein thrombosis			
Present	11 (33%)	13 (18%)	0·091
Absent	22 (67%)	58 (82%)	··
Cirrhosis			
Present	20 (61%)	26 (37%)	0·022
Absent	13 (39%)	45 (63%)	··
Previous locoregional treatment			
No	27 (82%)	56 (79%)	0·73
Yes	6 (18%)	15 (21%)	··
Tumour diameter, cm	7·1 (3·9–10·7)	6·0 (4·0–9·9)	0·45
Number of nodules			
Single	19 (58%)	52 (73%)	0·13
Multinodular	13 (39%)	18 (25%)	··
Data missing	1 (3%)	1 (1%)	··

Data are median (range) for age, median (IQR) for tumour diameter, and n (%) for all other variables.

## Data Availability

The dataset used in the study is not publicly available, but anonymised patient-level data may be available from the corresponding author upon written request within 24 months from the publication of the Article, depending on the policy and procedures of the individual trials and institutions that participate in the consortium. A detailed proposal for how the data will be used should be sent to the corresponding author and is required to allow for assessment of the application.

## Research in context

### Evidence before this study

We searched PubMed for studies published in the English language between Jan 1, 2020, and Feb 29, 2024, related to patients with hepatocellular carcinoma receiving immune checkpoint inhibitor (ICI) therapy before liver resection, using the following search terms: “hepatocellular carcinoma”, “immune checkpoint inhibitors”, “neoadjuvant”, “liver resection”, “pathological response”, and “clinical trials”. Although several clinical trials had investigated different types of ICIs before surgery, we found no multicentre study that had pooled patient-level data from different clinical trials to specifically investigate the role of pathological response in predicting relapse-free survival among patients with hepatocellular carcinoma.

### Added value of this study

To the best of our knowledge, this is the first study showing that the occurrence of pathological response after preoperative ICI-based therapy in patients with hepatocellular carcinoma is associated with relapse-free survival in a cross-trial cohort. By analysing a unique dataset of patient-level data from prospective clinical trials, we describe the characteristics of tumour regression and its association with long-term survival outcomes. Previous studies did not follow a consistent definition of major pathological response, leading to varying cutoffs for measuring tumour regression. This study is the first analysis across multiple trials to identify the optimal threshold of pathological response associated with a reduced risk of relapse.

### Implications of all the available evidence

Our study supports the use of pathological response as a predictor of long-term survival outcomes; however, its value as a surrogate endpoint for relapse-free survival should be validated in prospective randomised controlled trials. Our results inform the integration of ICI-based regimens in patients with hepatocellular carcinoma considered for liver resection. In light of the novel evidence on the use of adjuvant ICI combinations after liver resection, our study could help to identify patients with no pathological response after neoadjuvant ICI therapy as patients at increased risk of disease relapse.
