# Supplementary material for: Pathological response following neoadjuvant immune checkpoint inhibitors in patients with hepatocellular carcinoma: a cross-trial, patient-level analysis
Source: Lancet Oncol. Author manuscript; Available in PMC 2025 Apr 29. (PMC12040480; doi:10.1016/S1470-2045(24)00457-1)
Supplement: Supplement Appendix [file NIHMS2072699-supplement-Supplement_Appendix.pdf]

THE LANCET
Oncology

Supplementary appendix

This appendix formed part of the original submission and has been peer reviewed. We post it as supplied by the authors.

Supplement to: D'Alessio A, Stefanini B, Blanter J, et al. Pathological response following neoadjuvant immune checkpoint inhibitors in patients with hepatocellular carcinoma: a cross-trial, patient-level analysis. *Lancet Oncol* 2024; published online Oct 19. https://doi.org/10.1016/S1470-2045(24)00457-1.

Pathological response following neoadjuvant immune checkpoint inhibitors in patients with hepatocellular carcinoma: a cross-trial, patient-level analysis.

Antonio D'Alessio et al.

Supplementary Materials.

Table of Contents

SUPPLEMENTARY FIGURES.

Supplementary Figure 1. **Page 4.**
Representative haematoxylin/eosin-stained tissue sections from surgical samples of a patient showing no evidence of pathologic regression (A) and from a patient with complete pathological response (B). The tumour bed is identified with an asterisk.

Supplementary Figure 2. **Page 5.**
Kaplan-Meier curves representing the relapse-free survival (A) and the overall survival (B) of the whole cohort.

Supplementary Figure 3. **Page 6.**
Waterfall plots representing the depth of individual pathological responses across treatment modalities. Each patient is coloured differently according to their radiological response assessed per RECIST v1.1 criteria. A horizontal line represents the 70%-threshold for major pathological response (MPR). Abbreviations: PD-1, programmed death 1; mAb, monoclonal antibody; CTLA-4, cytotoxic T-lymphocyte associated protein 4; TKI, tyrosine-kinase inhibitor; PD-L1, programmed death ligand 1; VEGF, vascular endothelial growth factor.

Supplementary Figure 4. **Page 7.**
Kaplan-Meier curves representing the relapse-free survival according to the achievement of major pathological response (MPR) only in the population not receiving adjuvant treatment. Out of the 94 patients not treated with adjuvant immune checkpoint inhibitor in the study, we included in this analysis the 87 patients with an available MPR assessment. Abbreviations: HR, hazard ratio; p, p-value.

Supplementary Figure 5. **Page 8.**
Kaplan-Meier curves representing the relapse-free survival (RFS) according to the achievement of major pathological response (MPR) across presence of portal vein thrombosis (PVT), Barcelona Clinic Liver Cancer (BCLC) stage, and across treatment modality. Antiangiogenics encompasses tyrosine-kinase inhibitor (TKI) and anti-vascular endothelial factor (VEGF) Abbreviations: mRFS, median RFS; NR, not reached; NE, not estimable; p, p-value; PD-1, programmed death 1; CTLA-4, cytotoxic T-lymphocyte associated protein 4 ICI, immune checkpoint inhibitor.

Supplementary Figure 6. **Page 9.**
Graphical representations of Schoenfeld residuals method used to test the proportional hazard assumption for the variables included in the multivariable cluster-corrected Cox regression model and for the model as a whole.

Supplementary Figure 7. **Page 10.**
Kaplan-Meier curves representing the relapse-free survival according to the presence of baseline cirrhosis. Abbreviations: HR, hazard ratio; p, p-value.

Supplementary Figure 8. **Page 11.**
Graphical representation of the recursive partitioning of the tumour regression percentages showing the optimal cutoff of 90% to predict for the risk of relapse and/or death in the whole cohort (A). Panel B shows an additional recursive partitioning analysis adjusted per treatment centre and treatment modality, confirming the 90% cutoff. Panel C is a Receiver Operator Curve (ROC) where the continuous variable plotted is pathological response and the outcome of interest is relapse and/or death at 24 months. Abbreviations: p, p-value.

Supplementary Figure 9. **Page 12.**
Kaplan-Meier curves representing the overall survival according to the achievement of major pathological response (MPR) (A) and complete pathological response (pCR) (B). Abbreviations: HR, hazard ratio; p, p-value.

<u>SUPPLEMENTARY TABLES</u>.

Supplementary Table 1. **Page 13.**
Description of the clinical cohorts included in the NeoHCC consortium.

Supplementary Table 2. **Page 14.**
Radiological response assessed with Response Evaluation Criteria in Solid Tumours (RECIST) v1.1 criteria and modified RECIST (mRECIST) criteria.

Supplementary Table 3. **Page 15.**
Cluster-corrected Cox regression multivariable analysis for relapse-free survival (RFS).

Supplementary Table 4. **Page 16.**
Cluster-corrected Cox regression univariable analysis for relapse-free survival (RFS).

Supplementary Table 5. **Page 17.**
Description of baseline characteristics across 24-month relapse-free survival (RFS) status.

SUPPLEMENTARY METHODS.

Supplementary Methods 1. **Page 18.**
Description of the methods used for the assessment of pathological response.

Supplementary Figure 1.

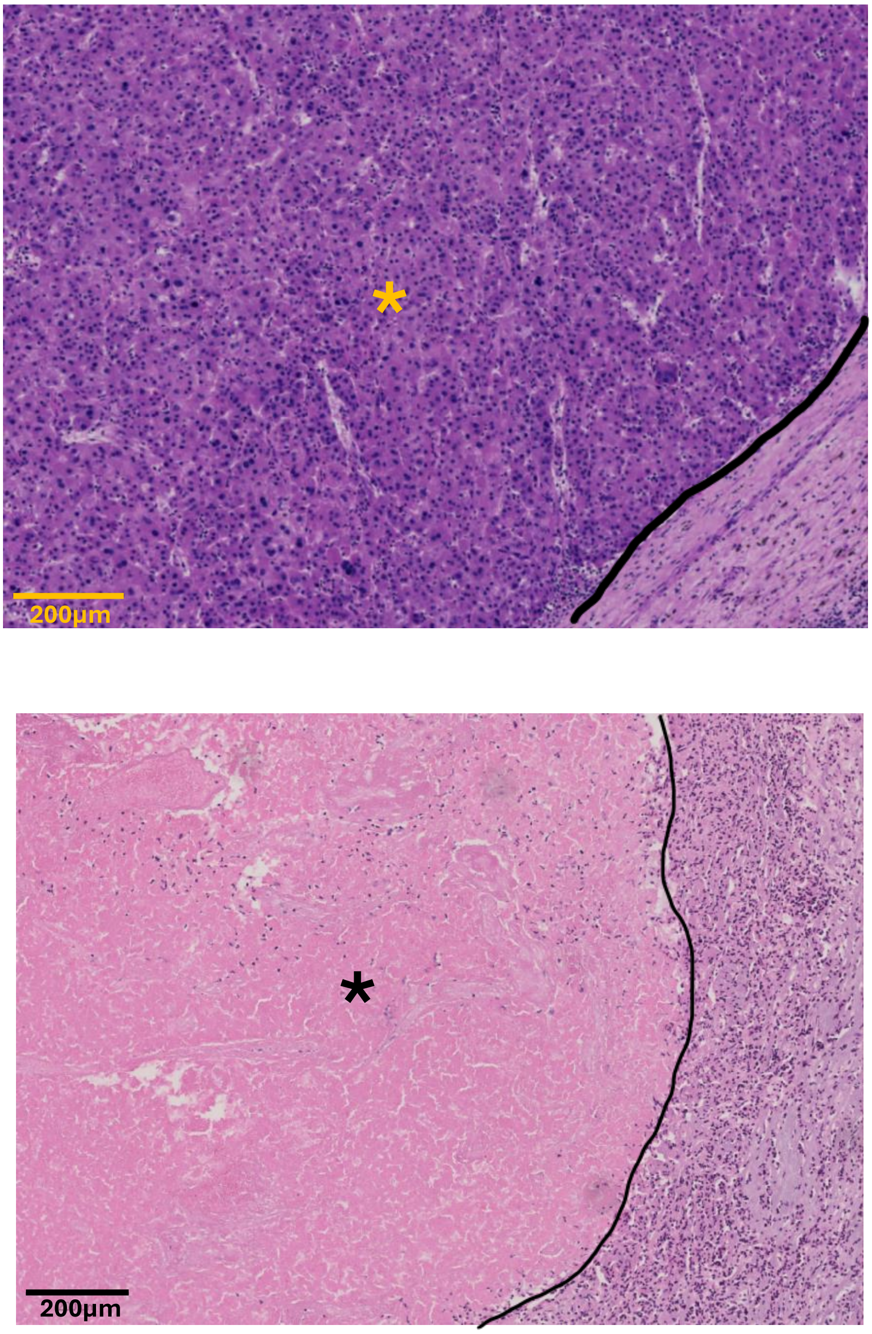

A

B

Supplementary Figure 2.

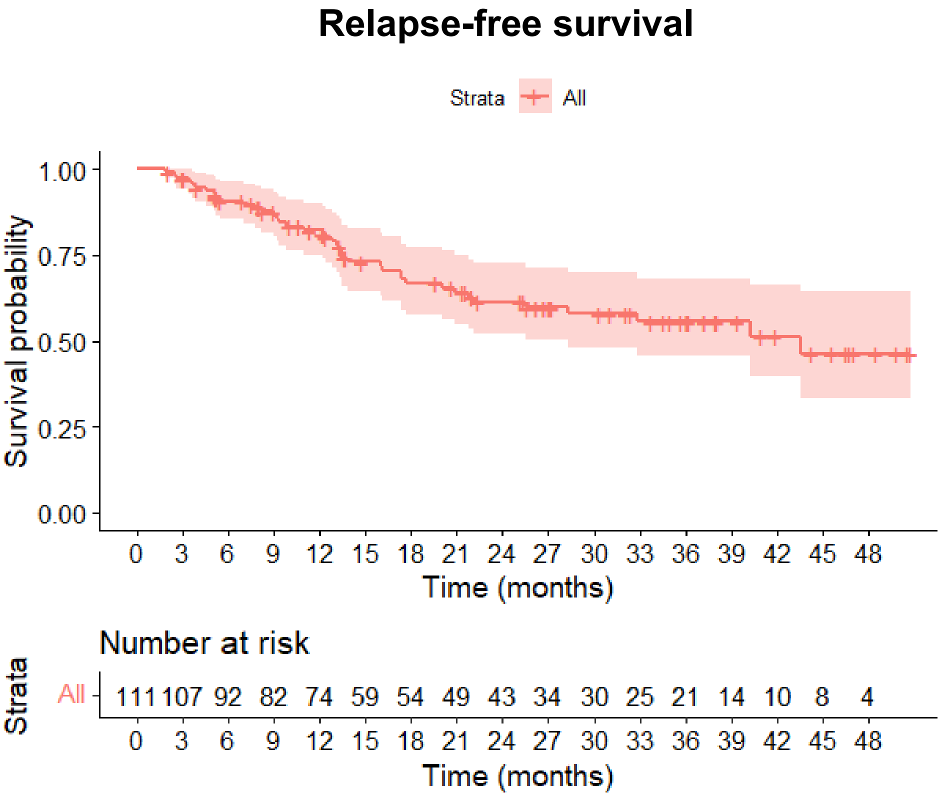

A

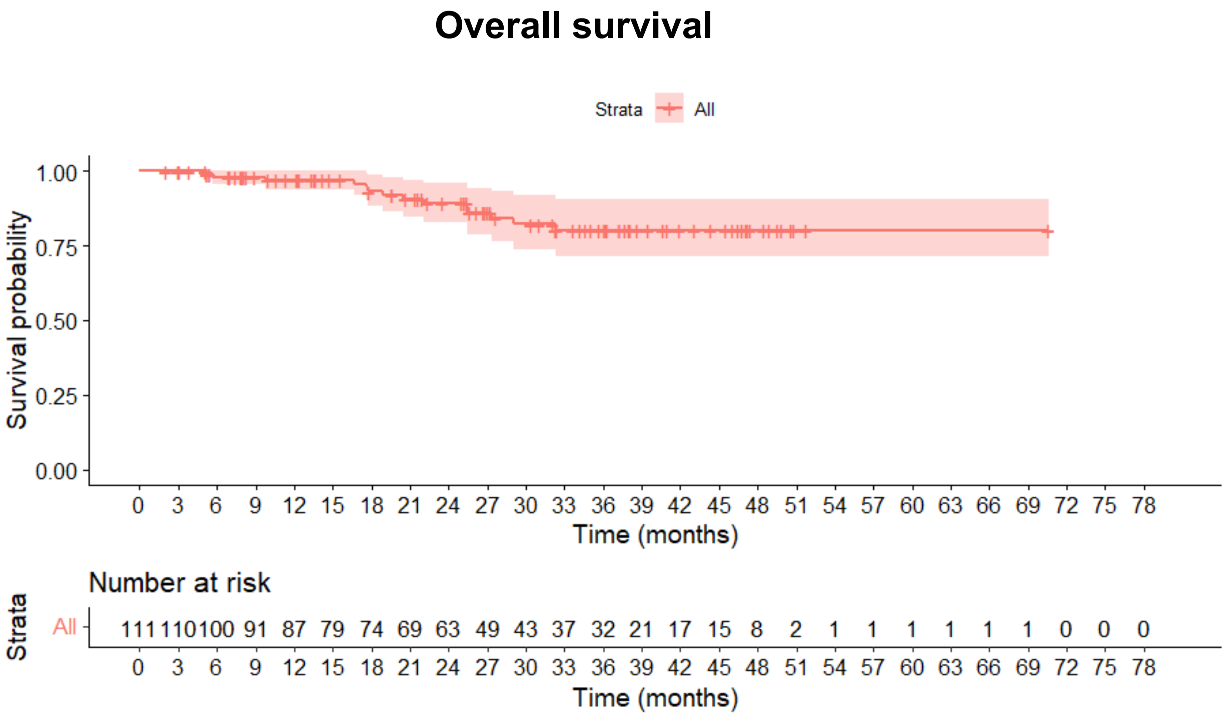

B

Supplementary Figure 3.

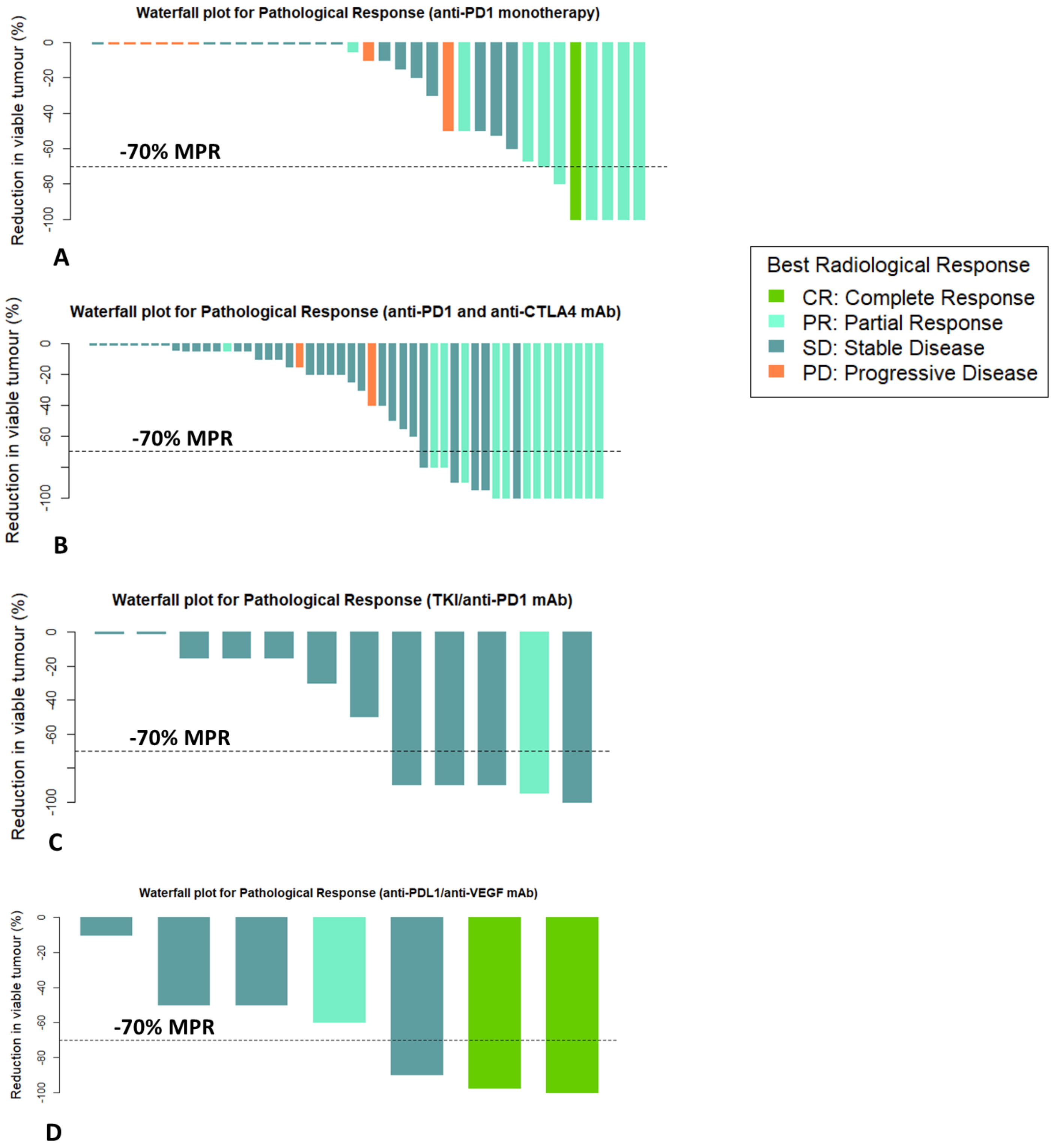

Supplementary Figure 4.

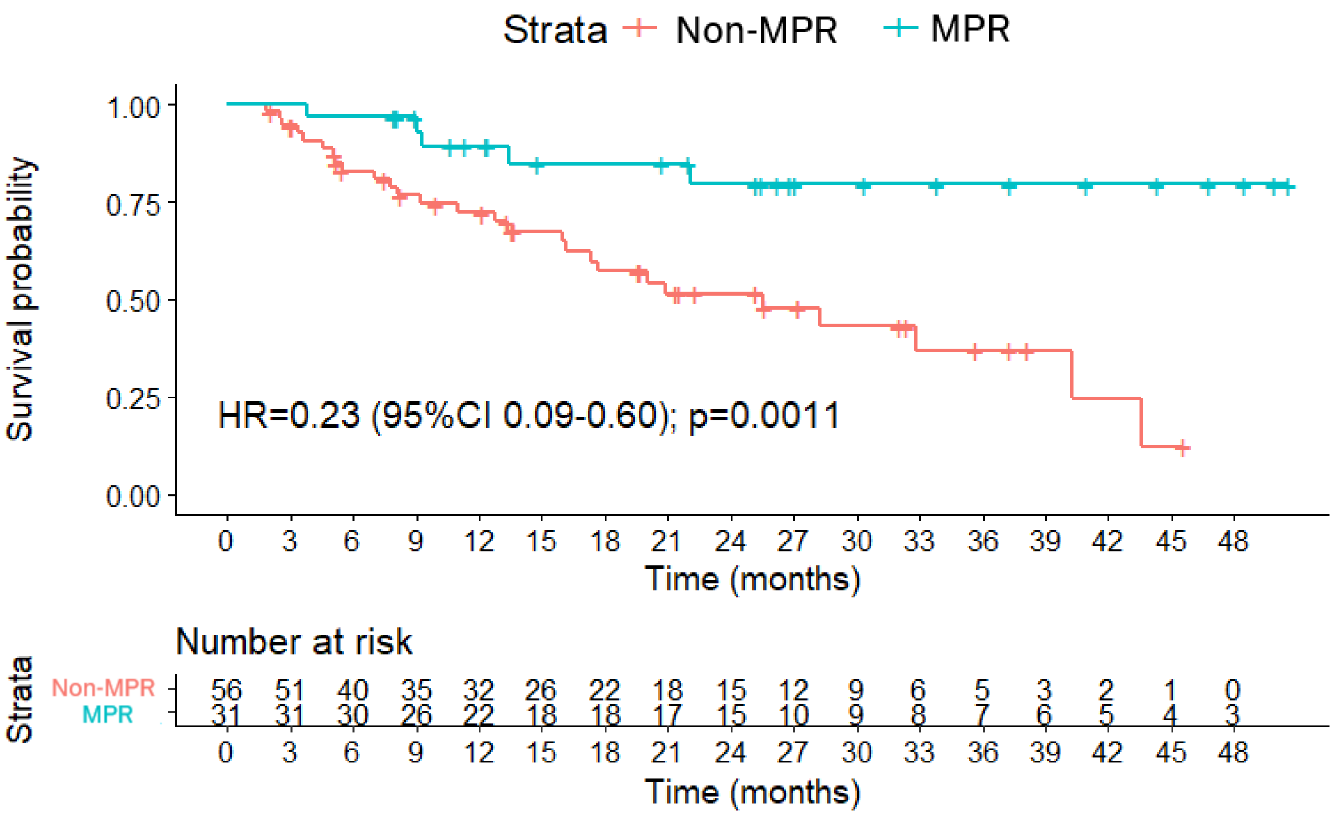

Supplementary Figure 5.

A

Patients without PVT

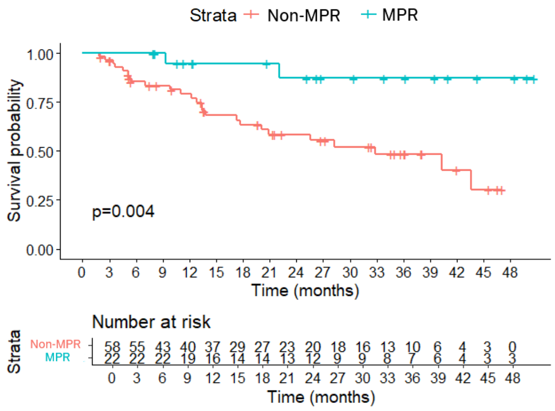

No PVT	Non-MPR	MPR	Log rank
mRFS in months (95%CI)	32.8 (20.1-NE)	NR	0.004

Patients with PVT

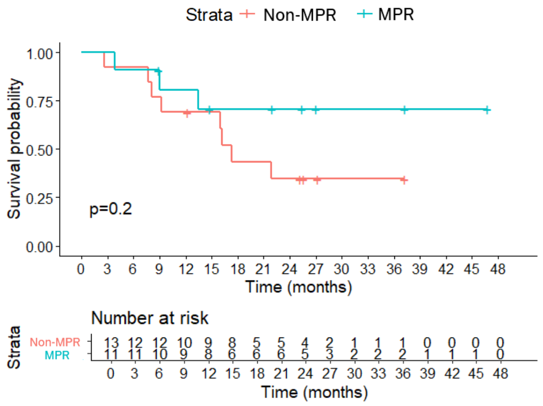

PVT	Non-MPR	MPR	Log rank
mRFS in months (95%CI)	17.3 (9.2-NE)	NR (13.4-NE)	0.2

B

BCLC A

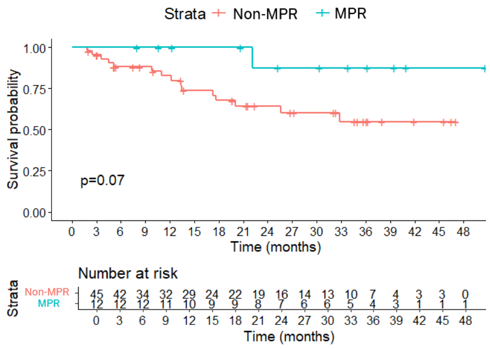

BCLC A	Non-MPR	MPR	Log rank
mRFS in months (95%CI)	NR (25.6-NE)	NR	0.07

BCLC B

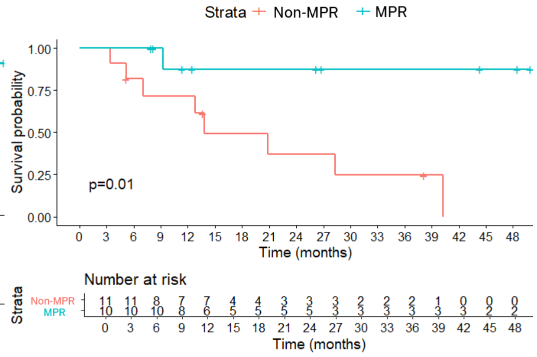

BCLC B	Non-MPR	MPR	Log rank
mRFS in months (95%CI)	13.8 (7.0-NE)	NR (NE-NE)	0.01

BCLC C

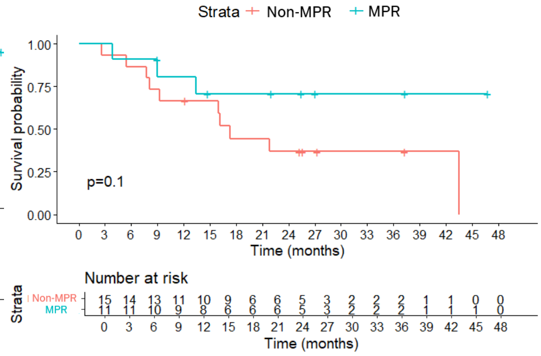

BCLC C	Non-MPR	MPR	Log rank
mRFS in months (95%CI)	17.3 (9.21-NE)	NR (13.4-NE)	0.1

C

Anti-PD-1/anti-CTLA-4

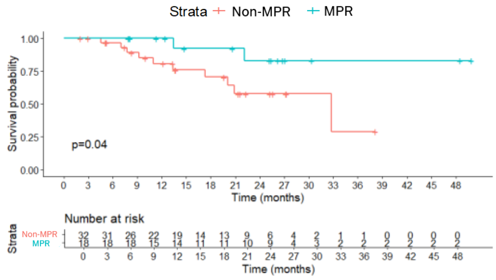

Double ICI	Non-MPR	MPR	Log rank
mRFS in months (95%CI)	32.8 (20.1-NE)	NR	0.04

ICI monotherapy

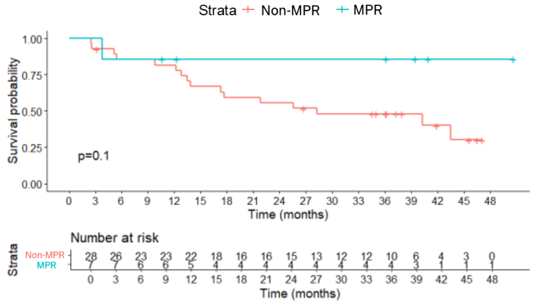

ICI monotherapy	Non-MPR	MPR	Log rank
mRFS in months (95%CI)	28.3 (17.1-NE)	NR	0.1

ICI + antiangiogenics

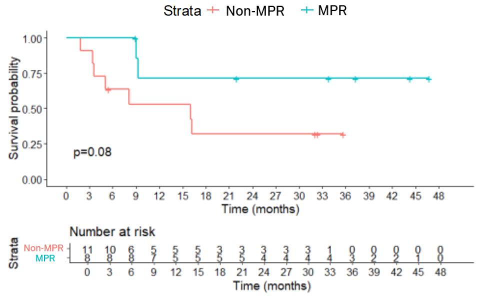

ICI/ antiangiogenics	Non-MPR	MPR	Log rank
mRFS in months (95%CI)	16.0 (15.1-NE)	NR (9.3-NE)	0.08

Supplementary Figure 6.

Variable	P value
Major Pathological Response	0.39
Sex	0.83
Aetiology	0.43
ECOG Performance Status	0.15
BCLC stage	0.78
Prior Locoregional Treatments	0.72
Cirrhosis	0.81
**GLOBAL**	**0.89**

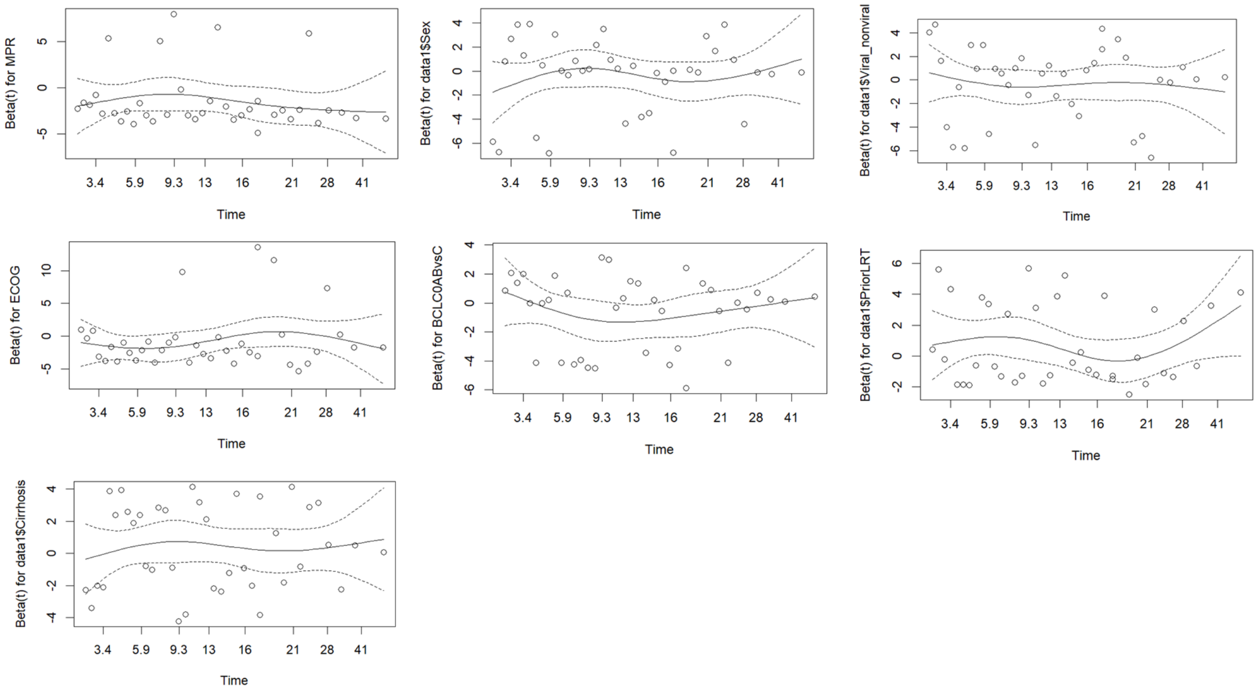

Supplementary Figure 7.

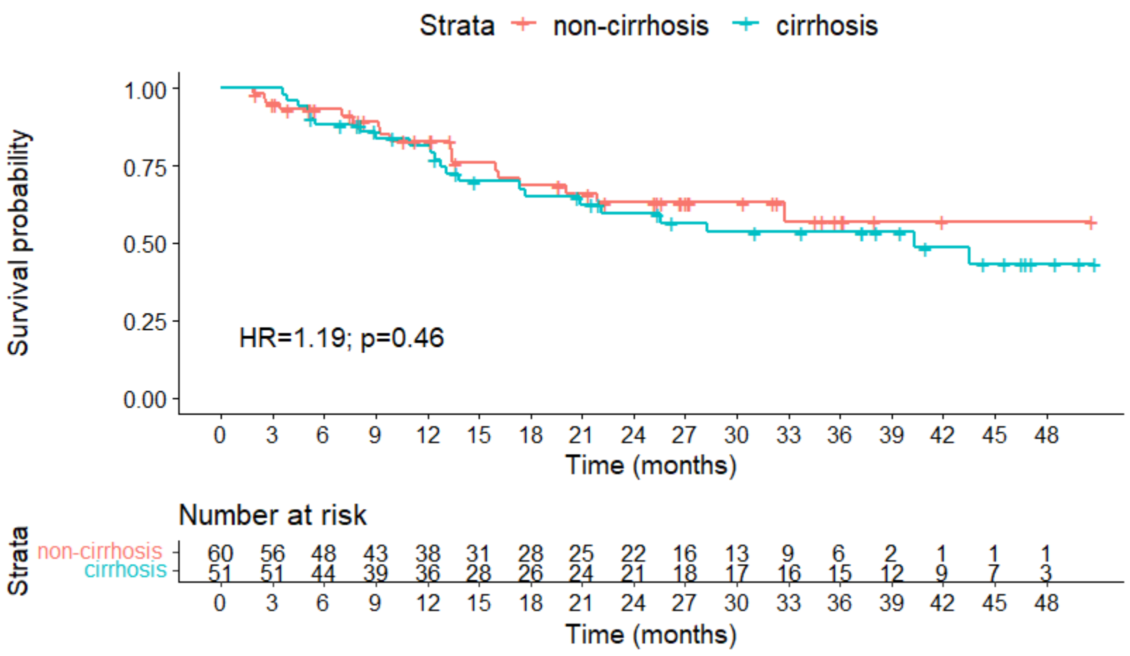

Supplementary Figure 8.

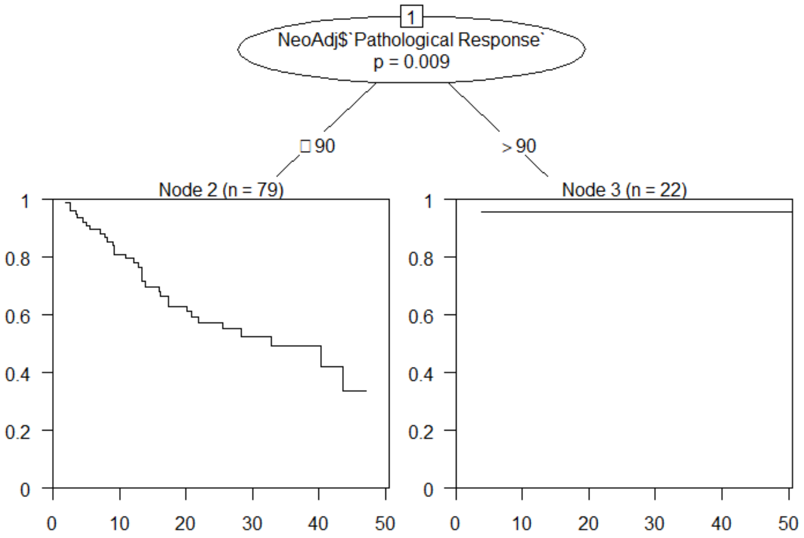

A

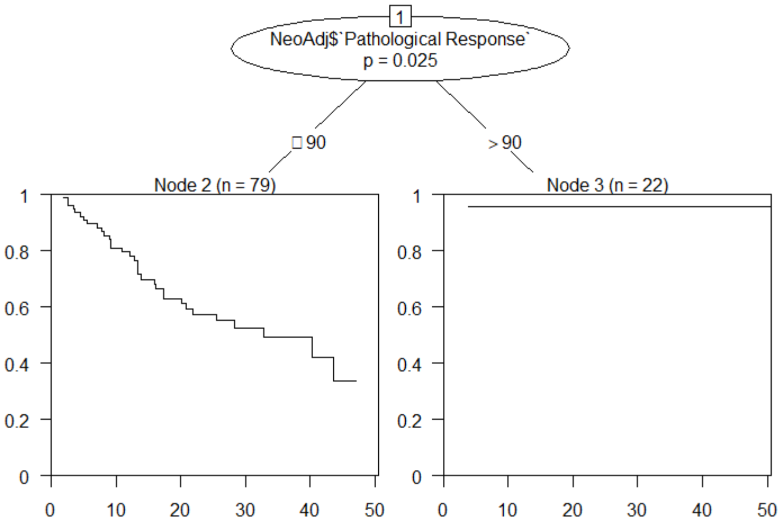

B

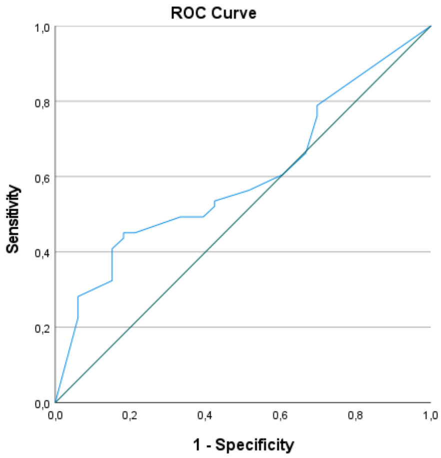

C

Area under the curve	95% Confidence Interval
0.60	0.49-0.71

Supplementary Figure 9.

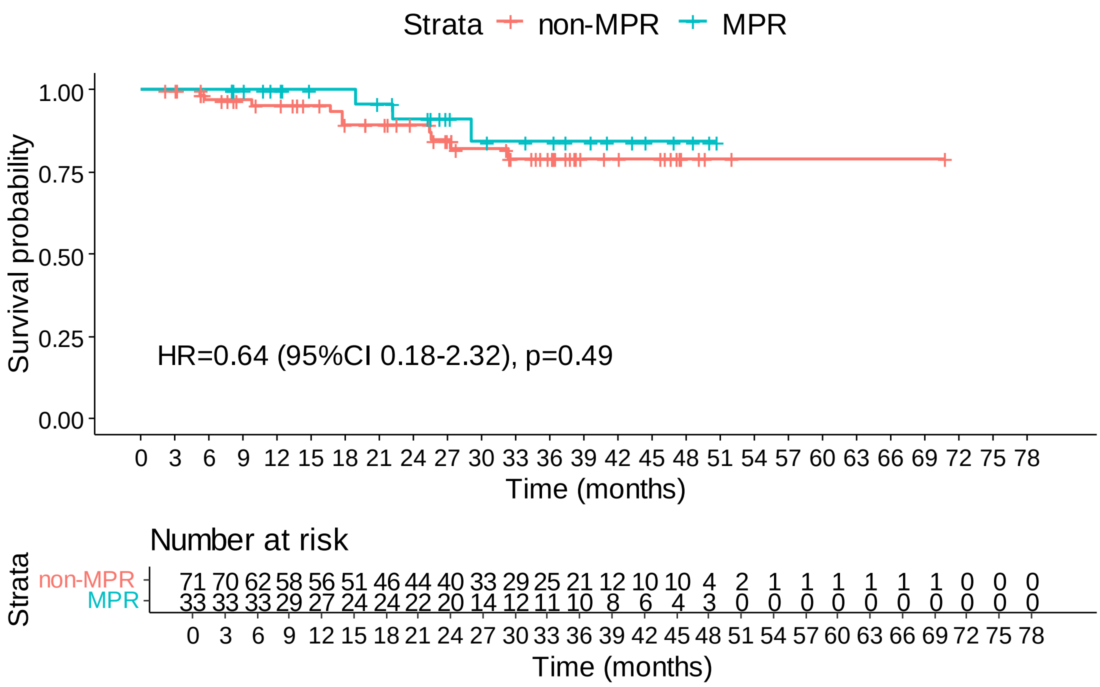

A

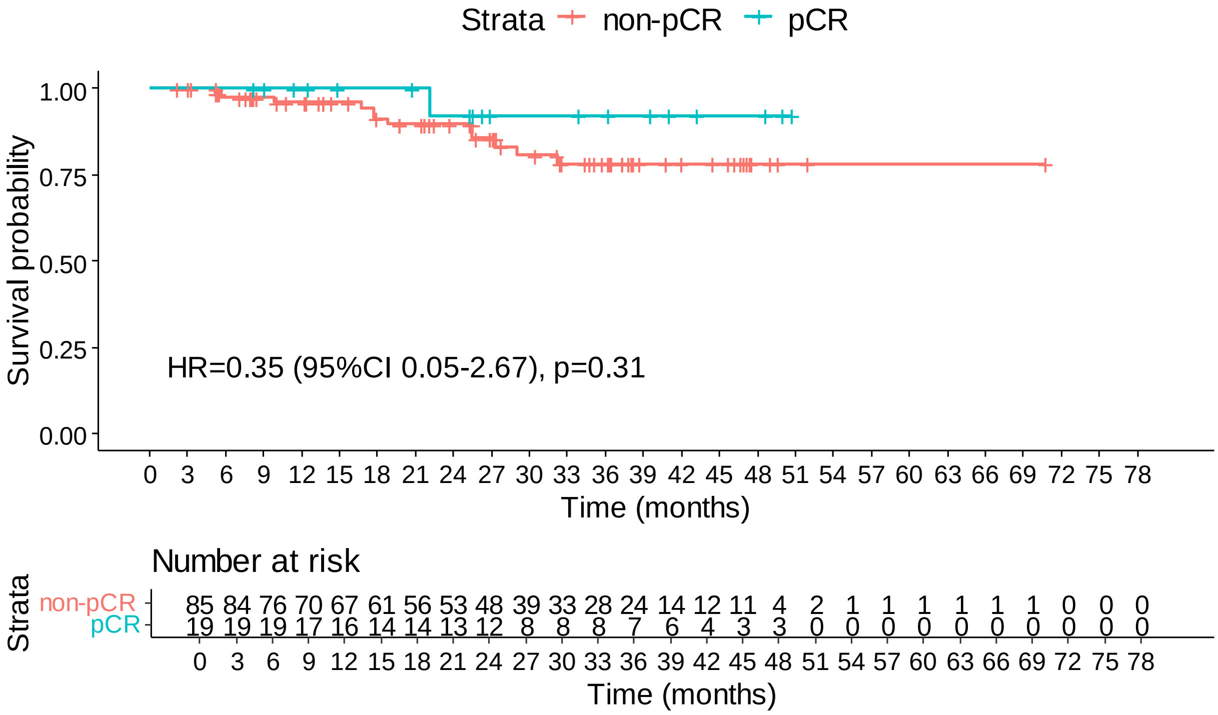

B

Supplementary Table 1. Description of the clinical cohorts included in the NeoHCC consortium.

Clinical Trial	Centres	Number of patients (total= 111)
**PRIME-HCC (NCT03682276)[12]**	Imperial College London (UK)<br>St Bartholomew's Hospital (UK)	27<br>6<br><br>**Total=33**
**NCT03510871[13]**	National Cheng Kung University Hospital<br>National Taiwan University Hospital<br>Chang Gung Memorial Hospital, Linkou<br>MacKay Memorial Hospital<br>Chang-Gung Memorial Hospital, Kaohsiung<br>Tri-Service General Hospital<br>Taipei Veterans General Hospital<br><br>**Taiwan Cooperative Oncology Group (TCOG)**	11<br>7<br>1<br>1<br>1<br>1<br>1<br><br>**Total=23**
**NCT03916627[8]**	Mount Sinai Hospital (US)	20
**NCT03299946[10]**	Johns Hopkins University (US)	12
**Observational clinical study[20]**	Mount Sinai Hospital (US)	23

Abbreviations: UK, United Kingdom; US, United States.

Supplementary Table 2. Radiological response assessed with Response Evaluation Criteria in Solid Tumours (RECIST) v1.1 criteria and modified RECIST (mRECIST) criteria.

	**RECIST v1.1 (n=111)**	**mRECIST (n=81)**
Complete Response	4% (n=4)	6% (n=5)
Partial Response	24% (n=27)	26% (n=21)
Stable Disease	63% (n=70)	63% (n=51)
Progressive Disease	9% (n=10)	5% (n=4)
**Overall Response Rate**	28% (n=31)	32% (n=26)
**Disease Control Rate**	91% (n=101)	95% (n=77)

Supplementary Table 3. Cluster-corrected Cox regression multivariable analysis for relapse-free survival (RFS).

	**HR 95%CI (P-value)**
MPR ***Y vs N***	0.24 0.15-0.38 **(<0.001)***
Sex ***M vs F***	0.66 0.27-1.62 (0.37)
Aetiology ***Viral vs Non Viral***	0.72 0.30-1.74 (0.47)
ECOG PS ***0 vs 1***	0.50 0.20-1.27 (0.14)
BCLC stage ***A/B vs C***	0.59 0.50-0.70 **(<0.001)***
Prior local therapy ***Y vs N***	1.93 1.72-2.16 **(<0.001)***
Cirrhosis ***Y vs N***	1.37 0.75-2.50 (0.30)

Abbreviations: HR, Hazard Ratio; CI, Confidence Interval; MPR, Major Pathological Response; Y, yes; N, no; M, male; F, female; ECOG PS, Eastern Cooperative Oncology Group Performance Status; BCLC, Barcelona Clinic Liver Cancer. *p value <0.05.

Supplementary Table 4. Cluster-corrected univariable analysis of the association between baseline characteristics and relapse-free survival.

	**HR 95%CI (P-value)**
**Sex** ***M vs F***	1.32 0.63-2.78 (0.47)
**Aetiology** ***Viral vs Non Viral***	1.17 0.60-2.28 (0.64)
**ECOG PS** ***0 vs 1***	0.71 0.25-2.00 (0.52)
**BCLC stage** ***A/B vs C***	0.67 0.35-1.27 (0.26)
**Prior local therapy** ***Y vs N***	2.29 1.69-3.09 **(<0.001)***
**Cirrhosis** ***Y vs N***	1.19 0.63-2.24 (0.59)
**Fibrosis** **F3/F4 vs F0/F1/F2**	1.34 0.94-1.91 (0.11)
**PVT** ***Y vs N***	1.43 0.83-2.46 (0.20)
**Maximum Tumour Diameter**	1.07 0.98-1.16 (0.11)
**Treatment modality** ***Combination ICI vs monotherapy***	0.82 0.63-1.07 (0.14)

Supplementary Table 5. Description of baseline characteristics and pathological response across 24-month RFS status.

Tot= 111 patients<br>Variable	Alive and free from relapse at 24 months (n=76)<br>N (%)	Relapse and/or death at 24 months (n=35)<br>N (%)	p-value
**Sex**			
Male	60 (79)	27 (77)	0.81
Female	16 (21)	8 (23)	
**ECOG PS**			
0	62 (82)	32 (91)	0.25
1	14 (18)	3 (9)	
**BCLC**			
A	45 (59)	16 (46)	0.22
B	18 (24)	8 (23)	
C	13 (17)	11 (31)	
**Etiology**			
Viral	50 (66)	23 (66)	1
Non-viral	26 (34)	12 (34)	
**PVT**			
Present	13 (17)	11 (31)	0.13
Absent	63 (83)	24 (69)	
**Cirrhosis**			
Present	33 (43)	18 (51)	0.54
Absent	43 (57)	17 (49)	
**Previous LRT**			
No	66 (87)	24 (69)	**0.035***
Yes	10 (13)	11 (31)	
**Fibrosis^**			
F0	12 (21)	3 (11)	0.71
F1	5 (9)	4 (15)	
F2	7 (12)	2 (8)	
F3	4 (7)	2 (8)	
F4	29 (51)	15 (58)	
**MPR §**			
Yes	28 (39)	5 (15)	**0.014***
No	43 (61)	28 (85)	

Abbreviations: ECOG PS, Eastern Cooperative Oncology Group Performance Status; BCLC, Barcelona Clinic Liver Cancer; PVT, Portal Vein Thrombosis; LRT, Locoregional Treatments; MPR, Major Pathological Response. *p value<0.05. ^28 missing values. §104 pathologically evaluable patients (n=71 alive and free from relapse at 24 months, n=33 relapsed and/or dead at 24 months).

Supplementary Methods 1.

Description of the methods used for the assessment of pathological response.

Tumour regression was assessed after histopathologic examination of all haematoxylin and eosin (H&E)-stained slides from post-ICI surgery containing tumour. The percentage of response resulted from the ratio of non-viable tumour (including necrotic component)/overall tumour bed x 100[1,2]. In case of multifocal tumours, we considered the mean of the pathological responses individually assessed for each tumour nodule. For patients with macrovascular invasion identified on pre-surgical imaging, assessment of pathological response included review of H&E-stained vascular sections. Each case was reviewed at each participating centre by a locally appointed study pathologist with at least 10 years of experience in liver histopathology and delegated by the principal investigator for the quantification of percentage of tumour regression. In one centre, a cohort of 23 patients was treated with neoadjuvant ICI as part of an observational protocol previously published[3], and pathological response was assessed using the same methodology of the clinical trial run in the same institution[4].

References.

1. Cottrell TR, Thompson ED, Forde PM, et al. Pathologic features of response to neoadjuvant anti-PD-1 in resected non-small-cell lung carcinoma: a proposal for quantitative immune-related pathologic response criteria (irPRC). *Ann Oncol* 2018; **29**(8): 1853-60.
2. Stein JE, Lipson EJ, Cottrell TR, et al. Pan-Tumor Pathologic Scoring of Response to PD-(L)1 Blockade. *Clin Cancer Res* 2020; **26**(3): 545-51.
3. Magen A, Hamon P, Fiaschi N, et al. Intratumoral dendritic cell-CD4(+) T helper cell niches enable CD8(+) T cell differentiation following PD-1 blockade in hepatocellular carcinoma. *Nat Med* 2023; **29**(6): 1389-99.
4. Marron TU, Fiel MI, Hamon P, et al. Neoadjuvant cemiplimab for resectable hepatocellular carcinoma: a single-arm, open-label, phase 2 trial. *The Lancet Gastroenterology & Hepatology* 2022; **7**(3): 219-29.
